# Immunosurveillance of Cancer and Viral Infections with Regard to Alterations of Human NK Cells Originating from Lifestyle and Aging

**DOI:** 10.3390/biomedicines9050557

**Published:** 2021-05-17

**Authors:** Xuewen Deng, Hiroshi Terunuma, Mie Nieda

**Affiliations:** 1 Biotherapy Institute of Japan, Inc., 2-4-8 Edagawa, Koto-ku, Tokyo 135-0051, Japan; terunuma@bij-net.com (H.T.); nieda.bij@gmail.com (M.N.); 2 N2 Clinic Yotsuya, 5F 2-6 Samon-cho, Shinjuku-ku, Tokyo 160-0017, Japan

**Keywords:** NK cell, immunosurveillance, lifestyle, aging, cancer, viral infection

## Abstract

Natural killer (NK) cells are cytotoxic immune cells with an innate capacity for eliminating cancer cells and virus- infected cells. NK cells are critical effector cells in the immunosurveillance of cancer and viral infections. Patients with low NK cell activity or NK cell deficiencies are predisposed to increased risks of cancer and severe viral infections. However, functional alterations of human NK cells are associated with lifestyles and aging. Personal lifestyles, such as cigarette smoking, alcohol consumption, stress, obesity, and aging are correlated with NK cell dysfunction, whereas adequate sleep, moderate exercise, forest bathing, and listening to music are associated with functional healthy NK cells. Therefore, adherence to a healthy lifestyle is essential and will be favorable for immunosurveillance of cancer and viral infections with healthy NK cells.

## 1. Introduction

The average global life expectancy has increased substantially over the past few decades [1]. The aging of the population has led to a high prevalence of chronic diseases such as cancer [2]. People with cancer have 5.8–11.2 years shorter life expectancy than their peers without cancer [3]. Lifestyles affect the incidence of chronic diseases including cancer and life expectancy [4]. Lifestyle factors including cigarette smoking, alcohol consumption, physical exercise, stress, obesity, sleep, forest bathing, and listening to music. Healthy lifestyles are related to a long life expectancy free from major diseases such as cancer [5] and higher resistance to viral infections. Conversely, unhealthy lifestyles contribute to the progression of severe diseases of such as COVID-19 [6] and increased risk of cancer progression [7]. Therefore, the promotion of a healthy lifestyle is critical to the defense against cancer and viral infections, and in improving life expectancy free of cancer and other associated chronic diseases [5]. In this paper, we review the immunosurveillance of cancer and viral infections with regard to alterations of natural killer (NK) cells originating from lifestyles and aging.

## 2. NK Cells

NK cells were first identified in 1975 from the observation of their natural capacity to kill tumor cells without prior in vivo sensitization [8,9]. NK cells originate from the bone marrow, and human NK cells comprise 5–20% of all lymphocytes and are defined phenotypically by their expression of CD56 and lack of CD3 expression. Two distinct populations of human NK cells could be identified, based upon their cell-surface density of CD56. The majority of human NK cells have low-density expression of CD56 (CD56^dim^) and expression high levels of CD16, which comprise about 90% of peripheral blood NK cells, whereas 10% of NK cells are CD56^bright^CD16^dim^ or CD56^bright^CD16^negative^. The CD56^dim^ subset is more naturally cytotoxic, whereas the CD56^bright^ subset, which is localized in lymphoid tissue has the capacity to produce abundant cytokines [10,11,12]. However, CD56^bright^ NK cells can also become cytotoxic upon appropriate activation and play roles in different disease states, such as cancer and infections [13]. NK cell functions are tightly regulated by a balance of activating and inhibitory germline-encoded receptors [14,15,16]. Healthy cells express major histocompatibility complex class I (MHC-I) molecules, which mark these cells as “self”. MHC-I molecules act as ligands for inhibitory receptors on NK cells and contribute to “self-tolerance”, by preventing the-killing of these healthy cells by NK cells [15,17]. The MHC-I-specific inhibitory receptors include killer cell immunoglobulin-like receptors (KIRs) and lectin-like CD94-NKG2A heterodimers [15,16].

## 3. NK Cell Immunosurveillance of Cancer and Viral Infections

Cancer cells and virus-infected cells, down regulate MHC-I expression, which impairs the engagement with inhibitory receptors on NK cells, and lowers the inhibitory signaling threshold [15,17,18,19]. In addition, cancer cells and virus- infected cells upregulate NK cell activity ligands, which in response to NK-cell -activating receptors, such as NKG2D, CD244, NKp30, and NKp46, induce signaling pathways that trigger NK cell responses [15,16]. The coactivation of these receptors overcomes the NK regulatory balance to mount an effective response against cancer cells and virus- infected cells [15]. NK cell activation can result in direct lysis of target cells, through cytotoxic degranulation by perforin and granzyme B [15], and the simultaneous production of inflammatory cytokines, such as interferon-γ and tumor necrosis factor-α, which activate other immune components including adaptive response components [17,20].

NK cells play key roles in host defense against viral infections [21,22]. In humans, NK cells are important to the innate immune response against members of the herpesvirus, poxvirus, and papillomavirus families [23,24]. Patients with identified NK cell deficiencies are predisposed to particularly severe, recurrent viral infection [24,25]. Mouse models provide additional evidence that NK cells critically help in the control of several viral infections, most notably murine cytomegalovirus, poxviruses, and influenza viruses [26,27].

**Figure 1:**
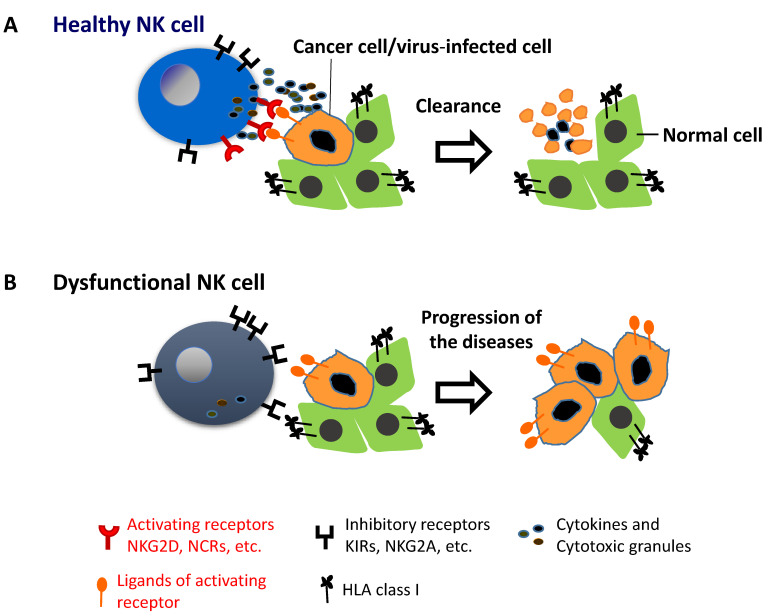
Schematic depiction of NK cell immunosurveillance of cancer and viral infections. (**A**): Healthy NK cells expressing activating receptors can recognize virus- infected cells and cancer cells, and release sufficient amount of cytotoxic granules and cytokines to kill and clear virus-infected cells and cancer cells. (**B**): Dysfunctional NK cells expressing an imbalance of activating and inhibitory receptors with high expression of inhibitory receptors, malfunctions in the recognition of cancer and virus-infected cells and the release of cytotoxic granules and cytokines to kill them, evasion of immunosurveillance of viral infections and cancer, and the spread of viral infections and progression of cancer.

Since their discovery, a numerous studies have demonstrated the NK-cell-mediated killing of many types of tumor cell line in vitro in experimental animal models [28,29,30,31,32,33]. NK cells contribute to the immunosurveillance of human cancer [34]. A prospective cohort study of 3625 people from the general population with 11-year follow up for cancer incidence and death indicated that people with medium or high NK cell activity had a reduced risk of cancer, whereas those with low NK cell activity manifested an increased cancer risk [35]. Additionally, patients with cancer, such as colorectal cancer [36], prostate cancer [37], and breast cancer [38] had decreased NK cell activity. Patients with metastatic cancer had furthermore decreased NK cell activity [38]. Several studies have demonstrated that a high degree of intratumoral infiltration of NK cells is associated with a favorable outcome [39,40,41]. Moreover, NK cell activity predicted the response to chemotherapy and immunotherapy for patients with cancer [42,43,44]. Therefore, functional healthy NK cells are critical for anti-tumor and anti-virus immunities, whereas NK cell dysfunction increases the vulnerability to viral infections and cancer (Figure 1).

## 4. Lifestyles and NK Cells

Owing to the unique capacities of NK cells in host immunoprotection, especially anti-tumor and anti-viral immunities, research data on NK cells in relation to lifestyles have accumulated over the past decades since the 1980s (Table 1). In this paper we provide an updated overview of the association of human NK cells with personal lifestyles, such as cigarette smoking, alcohol consumption, stress, obesity, sleep, exercise, forest bathing and listening to music. In particular, we examine findings of human studies on the alterations of NK cell count, percentage, subsets, phenotype, activity, cytokine secretion, and functional granule components in association with lifestyles.

### 4.1. Cigarette Smoking

Cigarette smoke is a major health risk factor that increases the risk of developing many types of cancer [83]. In a murine lung metastasis model, cigarette smoking impaired NK-cell-dependent tumor immunosurveillance, and the resulting altered immunity was associated with an increased tumor burden [84]. Studies in humans have shown that NK cell activity is reduced in smokers [45,46,47,48]. Recently, a large cross-sectional study has shown an inverse correlation between NK cell activity and the number cigarettes smoked daily, and the total number of cigarettes smoked [48]. Cigarette smoke reduces the cytolytic granules of perforin, granzymes, and granulysin on NK cells [49].

These finding suggest that cigarette smoking compromises NK cell function, enabling cancer cells to evade immunosurveillance.

### 4.2. Alcohol Consumption

Chronic heavy consumption of alcohol by humans has been implicated as an etiological factor or cofactor in various types of cancer [85]. Alcohol is known to have a generally suppressive effect on the immune system in humans [86,87]. However, research data about the effects of alcohol on NK cell activity are conflicting [50]. There are results indicating the NK cell count in the peripheral blood is increased [51] or decreased [52] by alcohol. Some results have shown an alcohol-related decrease in NK cell activity [53,54], while other studies did not find any significant effect of alcohol on NK cell activity [46,47,48]. A study of nonalcoholic male volunteers administered alcohol either intravenously or orally to has showed that a single dose did not alter NK cell activity even when the blood alcohol concentrations reached 89 mg/dl. However, when NK cells are exposed to alcohol in vitro for 4h at concentrations of 80 mg/dl and above, a significant concentration-dependent decrease in NK cell activity was observed [50]. Another study comparing the effects of alcohol on NK cells between patients with chronic alcoholism without liver disease (AWLD) and those with alcohol-induced cirrhosis (ALC) has shown that alcohol by itself induces an increase in the number and activity of NK cells in peripheral blood. In contrast, the NK cell activity is constantly depressed in the stage of alcoholic cirrhosis, suggesting that the behavior of NK cells in peripheral blood in chronic alcoholism differs depending on the presence or absence of ALC [51]. In addition, the level of perforin expression by NK cells is decreased in patients with chronic alcoholism [52]. The alterations of NK cells induced by chronic alcohol consumption, particularly in the stage of alcoholic cirrhosis, causes decreased immunosurveillance, which may contribute to the higher incidence of cancer.

### 4.3. Stress

Psychological stress affects various immune functions [88]. The hypothalamic-pituitary-adrenal (HPA) axis has been considered as a pathway along which psychological stress- is transposed into an impaired immune function [89]. NK cells express adrenergic receptors, which respond to catecholamines triggered by a stress response [90]. Subjects who complained of an unstable mental state have lower NK cell activity than those who reported a stable mental state [47]. Anxiety about cancer suppresses NK cell activity [55]. Stress levels in patients with breast cancer negatively correlated with NK cell activity [56]. Chronic job stress is associated with decreased NK cell count, proportion, and activity in peripheral blood [57]. People with job insecurity have decreased NK cell activity [58]. Stress depresses interferon (IFN) production by leukocytes concomitant with decreased NK cell activity [59]. Therefore, stress increases vulnerability to cancer and viral infections.

### 4.4. Obesity

Obesity increases the risk of many cancers and severe COVID-19 infection [91,92], and is responsible for up to 40% of cases of cancer [93]. Obesity drives chronic inflammation, which precedes the development of comorbid diseases including cancer [94]. NK cell anti-tumor responses are negatively regulated in obesity [95]. The number of NK cells is decreased in obese people [60,61], and is further decreased in obese people with unhealthy metabolic profiles [60]. There are increased CD56^bright^ NK subset and decreased CD56^dim^ subset in obese people, and correlates with body mass index (BMI) [62]. Obesity is associated with various alterations of the NK cell phenotype. Studies demonstrated that the expression level of the functional marker TRAIL and the activating NK receptor NKp46 are reduced in obese people [63,64,65]. In contrast, studies showed a highly activated NK cells in obese people with increased expression levels of CD69, and programmed cell death protein (PD)-1, as well as the inhibitory receptors of NKB1, CD158b, CD158i, NKG2A/CD94 complex, and Siglec-7 [60,61,63,65]. In addition, the expressions of CD16, and the adhesion molecule CD62L, and the maturation and differentiation marker CD27 on NK cells are downregulated in obese people [63]. Despite the enhanced activation, NK cells showed a decreased expression level of degranulating marker CD107a and decreased secretion of granzym B and perforin as well as the macrophage inflammatory protein (MIP)-1β in response to cancer cell lines in obese people [61,64,65]. Furthermore, the cytotoxicity of NK cells against cancer cells is significantly impaired in obese humans [61,63]. Aside from the characterization of total NK cells, the impact of obesity on the CD56^dim^ and CD56^bright^ subset-specific NK cell phenotype has recently been investigated, which demonstrated decreases in NKG2D-positive and CD69-positive CD56^dim^ NK cells, while there was an increase in NKG2D-positive CD56^bright^ NK cells in obese people. In addition, a study of cytokine production demonstrated a decreased expression level of IFN-γ positive CD56^dim^ NK cells, while there was an increased expression of IFN-γ positive CD56^bright^ NK cells in obese people. These alterations are correlated with BMI [62]. Therefore, obesity is associated with alterations of NK cell frequency and phenotypes and an impaired capacity to defend against cancer cells and virus-infected cells [61,63], which contribute to a higher cancer risk and susceptibility to viral infections in obese people. However, weight loss was found to reverse the obesity-induced defects in NK cells [62,96,97].

### 4.5. Sleep Deprivation

Sleep has a critical role in promoting health [98]. Sleep affects two primary effector systems, the HPA axis and the sympathetic nervous system (SNS), which in turn regulate immune responses [98,99]. NK cell activity is dependent in part on sleep. NK cell count and activity are minimum during the early part of the night and reach the maximum in the late morning hours [100]. There is a positive association between sleep time and efficiency with NK cell activity [101]. Loss of sleep at night resulted in decreases in both the number of NK cells and NK cell activity. After a night of recovery sleep, NK cell activity returned to baseline levels [66,67]. In addition, one night of sleep deprivation decreased NK activity [68]. In contrast, two nights of sleep deprivation increased NK activity [69]. The alterations of NK cell triggered by sleep loss were found to be mediated through augmented levels of glucocorticoids and catecholamine [99]. The resulting reduction in NK cell activity increased the vulnerability to cancer and viral infections.

### 4.6. Exercise

People who exercise almost daily have a reduced number of days of sickness [70,102]. Research has shown that positive immunological changes occur during moderate exercise [103]. NK cells have attracted the attention of exercise scientists for more than 30 years [104]. It has been reported that acute physical exercise strongly affects the NK cell count in peripheral blood [105]. The NK cell number increases immediately after cessation of exercise, followed by transient decreases in NK cell count and activity. Depending on the exercise regime (type, duration, and intensity), the decreases have been observed after 15–30 min and can persist for more than 24 h [105,106]. NK cells are rapidly mobilized into the circulation in response to acute exercise, most likely by epinephrine-dependent β–adrenergic signaling [107,108]. This mobilization primarily affects CD56^dim^ NK cells and is driven by the expression of adhesion molecules of CD11a and CX3CR1 [107,109]. In view of the physical fitness level, studies unanimously revealed an increased NK cell activity in subjects with good physical constitution [70,71,72,73,74]. NK cell count and activity are increased in racing cyclists compared with nonathletes [71]. Consistent with these findings, athletes were found to have higher NK cell count and activity [72,74]. People with exercise habits have higher counts of NK cells and perforin-, granulysin-, granzyme A/B- expressing NK cells [49]. Therefore, exercise habits strengthen the NK cell function to defend against cancer and viral infections.

### 4.7. Forest Bathing

A forest bathing trip is a short, leisurely visit to a forest and is regarded as being similar to natural aromatherapy. Incorporating forest bathing trips into a good lifestyle was first proposed in 1982 by the Forest Agency of Japan. The results of studies using the Profile of Mood States test demonstrated that a forest bathing trip significantly increased the score for vigor and decreased the scores for anxiety, depression, and anger [75,76]. Forest bathing stabilizes autonomic nervous activity and significantly decreases the concentrations of stress hormones such as adrenaline and noradrenaline [75,77,78]. The frequency and count of NK cells, and the expression levels of granulysin, perforin, and granzymes A/B on NK cells increased on the forest bathing days, resulting in an increase in NK cell activity [75,76,77,78,79,110]. The increased NK cell activity lasted for more than 30 days after the trip [75,77,79]. The mechanisms underlying the increased NK cell activity during forest bathing may be partially related to an attenuated stress hormone response and also to breathing in of volatile organic compounds, called phytoncides produced by trees, such as α–pinene and limonene [75,76,77,110]. In vitro research data indicate that phytoncides increase NK cell activity in a dose-dependent manner and prevent dichlovos-induced inhibition of NK cell activity [79]. Therefore, forest bathing trips have a positive effect on immunosurveillance against cancer and viral infections.

### 4.8. Music

Music therapy has attracted the attention of various fields such as psychiatry and geriatrics. Music therapy improves mood states by decreasing tension/anxiety, depression/dejection, anger/hostility, fatigue/inertia, and confusion/bewilderment and increases vigor/activity [80,81,111,112]. Music reduces the levels of stress hormones [113] and provides relief from stress [114,115]. The frequency of NK cells and NK cell activity are increased after music therapy [80,82]. Therefore, music provides a “wellness environment”, which improves defense against cancer and viral infections.

## 5. Aging with NK Cells

Aging is accompanied by dysregulated immune function that contributes to an increased susceptibility to diseases, such as cancer and infections [116]. Physiological aging is associated with changes in the composition, phenotype, and function of circulating NK cells [117]. Studies to date have shown the effects of aging on human NK cells (Table 2). Significant increases in the percentage and absolute number of NK cells are the general observations reported [118,119,120]. Regarding to NK cell subsets, studies have shown that the proportions and number of CD56^dim^ NK cells increases with age [118,120,121], and older adults possess significantly fewer CD56^bright^ NK cells [118,119,120,121], resulting in a marked age-related increase in the CD56^dim^:CD56^bright^ ratio [118,120,121]. Regarding the effects of aging on the NK cell phenotype, there is an age-related decrease in the expression level of activating receptors, such as NKp30 and/or NKp46 [118,120], and the expression levels of inhibitory receptors such as CD94 and/or NKG2A also show age-associated reductions [118,120,121]. Additionally, the proportion of CD57, a marker of NK maturity, is higher in older adults [120], whereas the expression level of CD69, an activation marker, increases with aging [119]. However, the increased expression level of CD57 on NK cells has been shown to be affected by infection by viruses, such as human cytomegalovirus [122]. CD57 is not necessarily a marker of aging/immunosenescence [123]. Importantly, aging is accompanied by decreased NK cell activity, which may be due to an age-associated impairment in perforin secretion [120]. Prospective studies have demonstrated that low NK cell activity is associated with an increased susceptibility to viral infections [124] and cancer [35].

## 6. Immunosurveillance of Cancer and Viral Infections by Healthy NK Cells

**Figure 2:**
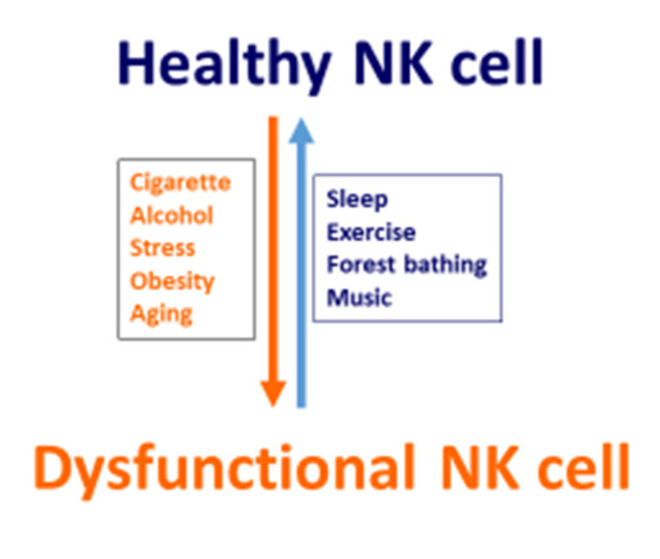
Schematic short list of the impacts of lifestyles and aging on NK cells. Cigarette smoking, alcohol consumption, stress, obesity, and aging suppress NK cell function, leading to a dysfunctional NK cells, whereas sleep, exercise, forest bathing, and listening to music enhance NK cell function with maintained functional healthy NK cells.

NK cells are lymphocytes of the innate immune system, which can deal promptly with stressed cells, such as cancer cells and virus-infected cells, while also regulating the body’s adaptive immunoresponses [125]. However, alterations in NK cells are associated with certain lifestyles [47,49,124,126] and aging [117]. Personal lifestyles, such as cigarette smoking, alcohol consumption, stress, obesity, and aging are associated with NK cell dysfunction, whereas adequate sleep, exercise, forest bathing, and listening to enjoyable music are associated with a healthy NK cell function (Figure 2). Therefore, adherence to a healthy lifestyle is essential and will be favorable to the immunosurveillance of cancer and viral infections with functional healthy NK cells.

## 7. Future Directions

Although there are many confounding factors for the associations among lifestyle, NK cells, and cancer or viral infections, future research is needed to provide scientific evidence of immunosurveillance of cancer and viral infections in relation to the alterations of NK cells caused by lifestyles and aging. Clinical studies are also necessary to clarify the importance of improving lifestyles in disease prevention.

For disease treatment using NK cells, NK-cell-based adoptive immunotherapy has been introduced for cancer treatment and has been considered for viral infections [19,127,128]. To obtain convincing evidence of the importance of NK cells for disease treatment, it is necessary to treat cancer or viral infections using the combination of NK cell therapy and lifestyle improvement. To this end, further studies are necessary.

## 8. Conclusions

NK cells play key roles in the innate elimination of cancer cells and virus-infected cells. Alterations of NK cells are associated with lifestyles and aging. Adherence to a healthy lifestyle is essential and will be favorable to the immunosurveillance of cancer and viral infections with healthy NK cells.

## Figures and Tables

**Table 1:** Overview of Lifestyles Associated Alterations of Human NK Cells.

	Study	NK Cells Count	NK Cell (%)	NK Cell Subsets	Receptors Expression on NK Cell	NK Activity	Chemokine and Cytokine Production	Functional Granule Components
Cigarette Smoke	Takeuchi M et al. [45]	N/A	N/A	N/A	N/A	↓	N/A	N/A
	Kusaka Y et al. [46]	N/A	N/A	N/A	N/A	↓	N/A	N/A
	Morimoto K et al. [47]	N/A	N/A	N/A	N/A	↓	N/A	N/A
	Jung YS et al. [48]	N/A	N/A	N/A	N/A	↓	N/A	N/A
	Li Q et al. [49]	↓	N/A	N/A	N/A	N/A	N/A	Perforin↓, GRN↓, GrA↓
Alcohol Consumption	Kusaka Y et al. [46]	N/A	N/A	N/A	N/A	↔	N/A	N/A
	Morimoto K et al. [47]	N/A	N/A	N/A	N/A	↔	N/A	N/A
	Li Q et al. [49]	↔	N/A	N/A	N/A	N/A	N/A	GrA↓
	Ochshorn-Adelson M et al. [50]	N/A	N/A	N/A	N/A	↓	N/A	N/A
	Laso FJ et al. [51]	↑	N/A	N/A	N/A	AWLD↑, ALC↓	N/A	N/A
	Perney P et al. [52]	↓	N/A	N/A	N/A	N/A	N/A	Perforin↓
	Redwine L, et al. [53]	N/A	N/A	N/A	N/A	↓	N/A	N/A
	Irwin M et al. [54]	N/A	N/A	N/A	N/A	↓	N/A	N/A
Stress	Morimoto K et al. [47]	N/A	N/A	N/A	N/A	↓	N/A	N/A
	Koga C et al. [55]	N/A	N/A	N/A	N/A	↓	N/A	N/A
	Andersen BL et al. [56]	N/A	N/A	N/A	N/A	↓	N/A	N/A
	Morikawa Y et al. [57]	↓	↓	N/A	N/A	↓	N/A	N/A
	Bosclo P et al. [58]	N/A	N/A	N/A	N/A	↓	N/A	N/A
	Glaser R et al. [59]	N/A	↓	N/A	N/A	↓	N/A	N/A
Obesity	Lynch LA et al. [60]	N/A	↓	N/A	NKB1↑, CD158b↑, CD69↑	N/A	N/A	N/A
	Tobin LM et al. [61]	↓	↓	N/A	CD69↑, PD-1↑, Glut-1↔	↓	IFN-γ↔	GrB↓, Perforin↓
	Bahr I et al. [62]	N/A	↔	CD56^bright^↑, CD56^dim^↓	CD56^bright^: NKG2D↑, CD56^dim^: NKG2D↓, CD69↓	N/A	CD56^bright^: IFN-γ↑, CD56^dim^: IFN-γ↓	N/A
	Naujoks W et al. [63]	N/A	↔	↔	NKp46↓, CD158i↓, NKG2A↑, Siglec-7↑, CD62L↓, CD27↓	↓	N/A	TRAIL↓
	Laue T et al. [64]	N/A	↔	↔	TRAIL↓, CD107a↓	N/A	N/A	N/A
	Viel S et al. [65]	↔	↔	N/A	NKp46↓, CD94↓, CD69↑, CD16↓, CD107a↓	N/A	IFN-γ↔, MIP1-β↓	GrB↑
Sleep deprivation	Irwin M et al. [66]	N/A	N/A	N/A	N/A	↓	N/A	N/A
	Irwin M et al. [67]	↓	↓	N/A	N/A	↓	N/A	N/A
	Moldofsky H et al. [68]	N/A	N/A	N/A	N/A	↓	N/A	N/A
	Dinges DF et al. [69]	↑	N/A	N/A	N/A	↑	N/A	N/A
Exercise	Li Q et al. [49]	↑	N/A	N/A	N/A	N/A	N/A	Perforin↑, GRN↑, GrA↑, GrB↑
	Nieman DC et al. [70]	↔	↑	N/A	N/A	↑	N/A	N/A
	Pedersen BK et al. [71]	N/A	↑	N/A	N/A	↑	N/A	N/A
	Moro-Garcia MA et al. [72]	N/A	↑	N/A	N/A	↑	N/A	N/A
	Jung YS et al. [73]	N/A	N/A	N/A	N/A	↑	N/A	N/A
	Nieman DC et al. [74]	↔	N/A	N/A	N/A	↑	N/A	N/A
Forest bathing	Li Q et al. [75]	N/A	↑	N/A	N/A	↑	N/A	Perforin↑, GRN↑, GrA↑,GrB↑
	Li Q et al. [76]	↑	↑	N/A	N/A	↑	N/A	Perforin↑, GRN↑, GrA↑, GrB↑
	Li Q et al. [77]	↑	↑	N/A	N/A	↑	N/A	Perforin↑, GRN↑, GrA↑, GrB↑
	Li Q et al. [78]	↑	↑	N/A	N/A	↑	N/A	Perforin↑, GRN↑, GrA↑,GrB↑
	Li Q [79]	↑	N/A	N/A	N/A	↑	N/A	Perforin↑, GrA↑, GrB↑
Music	Wachi M et al. [80]	N/A	↑	N/A	N/A	↑	IFN-γ↑, IL-10↓	N/A
	Koyama M et al. [81]	↔	N/A	N/A	N/A	↔	N/A	N/A
	Hasegawa Y et al. [82]	N/A	↑	N/A	N/A	↑	N/A	N/A

N/A: Not Available; ↔: No change; AWLD: Chronic Alcolism Without Liver Disease; ACL: Alcohol-Induced Cirrhosis. The up arrows “↑”, increase; down arrows “↓”, decrease.

**Table 2:** Overview of Aging Associated Alterations of Human NK Cells.

Study	NK Cells Count	NK Cell (%)	NK Cell Subsets	Receptors Expression on NK Cell	NK Activity	Chemokine and Cytokine Production	Functional Granule Components
Almeida-Oliveira A et al. [118]	↑	↑	CD56^bright^↓, CD56^dim^↑	NKp46↓, NKp30↓, CD94↓	N/A	N/A	N/A
Le Garff-Tavernier M, et al. [119]	↑	↔	CD56^bright^↓	CD69↑	↔	IFN-γ↔	N/A
Hazeldine J et al. [120]	N/A	↑	CD56^bright^↓, CD56^dim^↑	NKp46↓, CD94↓, CD57↑	↓	N/A	Perforin↓
Lutz CT et al. [121]	N/A	N/A	CD56^bright^↓, CD56^dim^↑	NKG2A↓	N/A	N/A	N/A

N/A: Not Available; ↔: No change. The up arrows “↑”, increase; down arrows “↓”, decrease.

## Data Availability

Data sharing not applicable.
